# Supplementary material for: Pyruvate Dehydrogenase Kinase 1 inhibition mediated oxidative phosphorylation enhancement in cartilage promotes osteoarthritis progression
Source: BMC Musculoskelet Disord. 2023 Jul 20;24:597. doi: 10.1186/s12891-023-06585-6 (PMC10357736; doi:10.1186/s12891-023-06585-6)
Supplement: Supplementary file 1 — Additional file 1. [file 12891_2023_6585_MOESM1_ESM.zip › MF.Gsea.1653627316242/GOMF_ACTIVE_TRANSMEMBRANE_TRANSPORTER_ACTIVITY.html]

Details for gene set GOMF\_ACTIVE\_TRANSMEMBRANE\_TRANSPORTER\_ACTIVITY[GSEA]

|  || Dataset | 55457\_collapsed\_to\_symbols.cls.cls #OA\_versus\_normal.cls.cls #OA\_versus\_normal\_repos |
| Phenotype | cls.cls#OA\_versus\_normal\_repos |
| Upregulated in class | OA |
| GeneSet | GOMF\_ACTIVE\_TRANSMEMBRANE\_TRANSPORTER\_ACTIVITY |
| Enrichment Score (ES) | 0.2230381 |
| Normalized Enrichment Score (NES) | 1.2114497 |
| Nominal p-value | 0.041825093 |
| FDR q-value | 0.52062136 |
| FWER p-Value | 1.0 |
Table: GSEA Results Summary

  

Fig 1: Enrichment plot: GOMF\_ACTIVE\_TRANSMEMBRANE\_TRANSPORTER\_ACTIVITY      
 Profile of the Running ES Score & Positions of GeneSet Members on the Rank Ordered List

  

| SYMBOL | TITLE | RANK IN GENE LIST | RANK METRIC SCORE | RUNNING ES | CORE ENRICHMENT || 1 | TMCO3 | transmembrane and coiled-coil domains 3 [Source:HGNC Symbol;Acc:HGNC:20329] | 12 | 1.165 | 0.0107 | Yes |
| 2 | ATP6V1C1 | ATPase H+ transporting V1 subunit C1 [Source:HGNC Symbol;Acc:HGNC:856] | 17 | 1.125 | 0.0215 | Yes |
| 3 | UQCR10 | "ubiquinol-cytochrome c reductase, complex III subunit X [Source:HGNC Symbol;Acc:HGNC:30863]" | 53 | 0.987 | 0.0286 | Yes |
| 4 | NDUFA8 | NADH:ubiquinone oxidoreductase subunit A8 [Source:HGNC Symbol;Acc:HGNC:7692] | 67 | 0.961 | 0.0371 | Yes |
| 5 | ATP6V0D1 | ATPase H+ transporting V0 subunit d1 [Source:HGNC Symbol;Acc:HGNC:13724] | 135 | 0.872 | 0.0405 | Yes |
| 6 | SLC22A18 | solute carrier family 22 member 18 [Source:HGNC Symbol;Acc:HGNC:10964] | 152 | 0.858 | 0.0478 | Yes |
| 7 | NDUFS3 | NADH:ubiquinone oxidoreductase core subunit S3 [Source:HGNC Symbol;Acc:HGNC:7710] | 177 | 0.836 | 0.0542 | Yes |
| 8 | NDUFA1 | NADH:ubiquinone oxidoreductase subunit A1 [Source:HGNC Symbol;Acc:HGNC:7683] | 194 | 0.826 | 0.0612 | Yes |
| 9 | RALBP1 | ralA binding protein 1 [Source:HGNC Symbol;Acc:HGNC:9841] | 217 | 0.808 | 0.0675 | Yes |
| 10 | NDUFA13 | NADH:ubiquinone oxidoreductase subunit A13 [Source:HGNC Symbol;Acc:HGNC:17194] | 236 | 0.795 | 0.0740 | Yes |
| 11 | NDUFA10 | NADH:ubiquinone oxidoreductase subunit A10 [Source:HGNC Symbol;Acc:HGNC:7684] | 243 | 0.788 | 0.0813 | Yes |
| 12 | NDUFB6 | NADH:ubiquinone oxidoreductase subunit B6 [Source:HGNC Symbol;Acc:HGNC:7701] | 262 | 0.779 | 0.0877 | Yes |
| 13 | ATP6V1E1 | ATPase H+ transporting V1 subunit E1 [Source:HGNC Symbol;Acc:HGNC:857] | 287 | 0.758 | 0.0933 | Yes |
| 14 | SLC5A3 | solute carrier family 5 member 3 [Source:HGNC Symbol;Acc:HGNC:11038] | 325 | 0.734 | 0.0977 | Yes |
| 15 | ANKH | ANKH inorganic pyrophosphate transport regulator [Source:HGNC Symbol;Acc:HGNC:15492] | 358 | 0.721 | 0.1023 | Yes |
| 16 | SLC25A31 | solute carrier family 25 member 31 [Source:HGNC Symbol;Acc:HGNC:25319] | 395 | 0.708 | 0.1065 | Yes |
| 17 | UQCRC1 | ubiquinol-cytochrome c reductase core protein 1 [Source:HGNC Symbol;Acc:HGNC:12585] | 473 | 0.672 | 0.1071 | Yes |
| 18 | NDUFB3 | NADH:ubiquinone oxidoreductase subunit B3 [Source:HGNC Symbol;Acc:HGNC:7698] | 483 | 0.667 | 0.1130 | Yes |
| 19 | ATP6V1G1 | ATPase H+ transporting V1 subunit G1 [Source:HGNC Symbol;Acc:HGNC:864] | 515 | 0.655 | 0.1171 | Yes |
| 20 | NDUFS4 | NADH:ubiquinone oxidoreductase subunit S4 [Source:HGNC Symbol;Acc:HGNC:7711] | 531 | 0.647 | 0.1224 | Yes |
| 21 | ATP6V0E2 | ATPase H+ transporting V0 subunit e2 [Source:HGNC Symbol;Acc:HGNC:21723] | 532 | 0.647 | 0.1288 | Yes |
| 22 | NDUFA3 | NADH:ubiquinone oxidoreductase subunit A3 [Source:HGNC Symbol;Acc:HGNC:7686] | 550 | 0.641 | 0.1338 | Yes |
| 23 | COX5B | cytochrome c oxidase subunit 5B [Source:HGNC Symbol;Acc:HGNC:2269] | 585 | 0.628 | 0.1374 | Yes |
| 24 | NDUFA4 | NDUFA4 mitochondrial complex associated [Source:HGNC Symbol;Acc:HGNC:7687] | 624 | 0.613 | 0.1405 | Yes |
| 25 | COX6A1 | cytochrome c oxidase subunit 6A1 [Source:HGNC Symbol;Acc:HGNC:2277] | 653 | 0.605 | 0.1443 | Yes |
| 26 | ABCC6 | ATP binding cassette subfamily C member 6 [Source:HGNC Symbol;Acc:HGNC:57] | 729 | 0.587 | 0.1442 | Yes |
| 27 | ATP6V1D | ATPase H+ transporting V1 subunit D [Source:HGNC Symbol;Acc:HGNC:13527] | 750 | 0.581 | 0.1484 | Yes |
| 28 | SLC2A10 | solute carrier family 2 member 10 [Source:HGNC Symbol;Acc:HGNC:13444] | 874 | 0.550 | 0.1441 | Yes |
| 29 | NDUFA2 | NADH:ubiquinone oxidoreductase subunit A2 [Source:HGNC Symbol;Acc:HGNC:7685] | 878 | 0.549 | 0.1494 | Yes |
| 30 | NDUFB5 | NADH:ubiquinone oxidoreductase subunit B5 [Source:HGNC Symbol;Acc:HGNC:7700] | 902 | 0.542 | 0.1529 | Yes |
| 31 | NDUFC2 | NADH:ubiquinone oxidoreductase subunit C2 [Source:HGNC Symbol;Acc:HGNC:7706] | 929 | 0.532 | 0.1562 | Yes |
| 32 | SLC18A2 | solute carrier family 18 member A2 [Source:HGNC Symbol;Acc:HGNC:10935] | 940 | 0.530 | 0.1607 | Yes |
| 33 | NDUFA9 | NADH:ubiquinone oxidoreductase subunit A9 [Source:HGNC Symbol;Acc:HGNC:7693] | 943 | 0.529 | 0.1658 | Yes |
| 34 | SLC10A3 | solute carrier family 10 member 3 [Source:HGNC Symbol;Acc:HGNC:22979] | 959 | 0.526 | 0.1698 | Yes |
| 35 | NDUFB4 | NADH:ubiquinone oxidoreductase subunit B4 [Source:HGNC Symbol;Acc:HGNC:7699] | 968 | 0.525 | 0.1744 | Yes |
| 36 | SLC9A6 | solute carrier family 9 member A6 [Source:HGNC Symbol;Acc:HGNC:11079] | 981 | 0.522 | 0.1787 | Yes |
| 37 | NDUFB7 | NADH:ubiquinone oxidoreductase subunit B7 [Source:HGNC Symbol;Acc:HGNC:7702] | 1033 | 0.510 | 0.1797 | Yes |
| 38 | NDUFB1 | NADH:ubiquinone oxidoreductase subunit B1 [Source:HGNC Symbol;Acc:HGNC:7695] | 1055 | 0.506 | 0.1831 | Yes |
| 39 | NDUFS7 | NADH:ubiquinone oxidoreductase core subunit S7 [Source:HGNC Symbol;Acc:HGNC:7714] | 1071 | 0.501 | 0.1869 | Yes |
| 40 | NDUFC1 | NADH:ubiquinone oxidoreductase subunit C1 [Source:HGNC Symbol;Acc:HGNC:7705] | 1091 | 0.499 | 0.1903 | Yes |
| 41 | NDUFA7 | NADH:ubiquinone oxidoreductase subunit A7 [Source:HGNC Symbol;Acc:HGNC:7691] | 1097 | 0.496 | 0.1949 | Yes |
| 42 | COX8A | cytochrome c oxidase subunit 8A [Source:HGNC Symbol;Acc:HGNC:2294] | 1105 | 0.494 | 0.1993 | Yes |
| 43 | ATP1B1 | ATPase Na+/K+ transporting subunit beta 1 [Source:HGNC Symbol;Acc:HGNC:804] | 1177 | 0.481 | 0.1984 | Yes |
| 44 | ATP6V0E1 | ATPase H+ transporting V0 subunit e1 [Source:HGNC Symbol;Acc:HGNC:863] | 1207 | 0.474 | 0.2008 | Yes |
| 45 | NDUFS8 | NADH:ubiquinone oxidoreductase core subunit S8 [Source:HGNC Symbol;Acc:HGNC:7715] | 1208 | 0.474 | 0.2056 | Yes |
| 46 | NDUFS1 | NADH:ubiquinone oxidoreductase core subunit S1 [Source:HGNC Symbol;Acc:HGNC:7707] | 1229 | 0.471 | 0.2087 | Yes |
| 47 | SLC35E2B | solute carrier family 35 member E2B [Source:HGNC Symbol;Acc:HGNC:33941] | 1302 | 0.458 | 0.2075 | Yes |
| 48 | ATP6V1H | ATPase H+ transporting V1 subunit H [Source:HGNC Symbol;Acc:HGNC:18303] | 1304 | 0.457 | 0.2120 | Yes |
| 49 | SLC1A4 | solute carrier family 1 member 4 [Source:HGNC Symbol;Acc:HGNC:10942] | 1372 | 0.447 | 0.2111 | Yes |
| 50 | NDUFS6 | NADH:ubiquinone oxidoreductase subunit S6 [Source:HGNC Symbol;Acc:HGNC:7713] | 1417 | 0.441 | 0.2120 | Yes |
| 51 | SLC25A11 | solute carrier family 25 member 11 [Source:HGNC Symbol;Acc:HGNC:10981] | 1448 | 0.436 | 0.2140 | Yes |
| 52 | CLCN3 | chloride voltage-gated channel 3 [Source:HGNC Symbol;Acc:HGNC:2021] | 1535 | 0.423 | 0.2114 | Yes |
| 53 | SLC2A9 | solute carrier family 2 member 9 [Source:HGNC Symbol;Acc:HGNC:13446] | 1634 | 0.408 | 0.2077 | Yes |
| 54 | NDUFV2 | NADH:ubiquinone oxidoreductase core subunit V2 [Source:HGNC Symbol;Acc:HGNC:7717] | 1642 | 0.407 | 0.2112 | Yes |
| 55 | SLC24A3 | solute carrier family 24 member 3 [Source:HGNC Symbol;Acc:HGNC:10977] | 1654 | 0.406 | 0.2143 | Yes |
| 56 | NDUFV1 | NADH:ubiquinone oxidoreductase core subunit V1 [Source:HGNC Symbol;Acc:HGNC:7716] | 1663 | 0.404 | 0.2177 | Yes |
| 57 | ABCG1 | ATP binding cassette subfamily G member 1 [Source:HGNC Symbol;Acc:HGNC:73] | 1711 | 0.396 | 0.2180 | Yes |
| 58 | SLC35A1 | solute carrier family 35 member A1 [Source:HGNC Symbol;Acc:HGNC:11021] | 1779 | 0.387 | 0.2165 | Yes |
| 59 | SURF1 | SURF1 cytochrome c oxidase assembly factor [Source:HGNC Symbol;Acc:HGNC:11474] | 1829 | 0.381 | 0.2164 | Yes |
| 60 | SLCO1A2 | solute carrier organic anion transporter family member 1A2 [Source:HGNC Symbol;Acc:HGNC:10956] | 1853 | 0.379 | 0.2183 | Yes |
| 61 | SLC34A2 | solute carrier family 34 member 2 [Source:HGNC Symbol;Acc:HGNC:11020] | 1994 | 0.362 | 0.2108 | Yes |
| 62 | CYB561D2 | cytochrome b561 family member D2 [Source:HGNC Symbol;Acc:HGNC:30253] | 2010 | 0.360 | 0.2132 | Yes |
| 63 | COX7A1 | cytochrome c oxidase subunit 7A1 [Source:HGNC Symbol;Acc:HGNC:2287] | 2045 | 0.356 | 0.2141 | Yes |
| 64 | SLCO1B1 | solute carrier organic anion transporter family member 1B1 [Source:HGNC Symbol;Acc:HGNC:10959] | 2051 | 0.356 | 0.2172 | Yes |
| 65 | SLC9A3 | solute carrier family 9 member A3 [Source:HGNC Symbol;Acc:HGNC:11073] | 2148 | 0.344 | 0.2130 | Yes |
| 66 | COX4I1 | cytochrome c oxidase subunit 4I1 [Source:HGNC Symbol;Acc:HGNC:2265] | 2167 | 0.342 | 0.2150 | Yes |
| 67 | SLC25A30 | solute carrier family 25 member 30 [Source:HGNC Symbol;Acc:HGNC:27371] | 2229 | 0.336 | 0.2135 | Yes |
| 68 | NDUFS5 | NADH:ubiquinone oxidoreductase subunit S5 [Source:HGNC Symbol;Acc:HGNC:7712] | 2267 | 0.330 | 0.2139 | Yes |
| 69 | ATP12A | ATPase H+/K+ transporting non-gastric alpha2 subunit [Source:HGNC Symbol;Acc:HGNC:13816] | 2330 | 0.323 | 0.2122 | Yes |
| 70 | NDUFB2 | NADH:ubiquinone oxidoreductase subunit B2 [Source:HGNC Symbol;Acc:HGNC:7697] | 2460 | 0.307 | 0.2050 | Yes |
| 71 | CLCN4 | chloride voltage-gated channel 4 [Source:HGNC Symbol;Acc:HGNC:2022] | 2498 | 0.303 | 0.2051 | Yes |
| 72 | COX7A2L | cytochrome c oxidase subunit 7A2 like [Source:HGNC Symbol;Acc:HGNC:2289] | 2517 | 0.302 | 0.2067 | Yes |
| 73 | SLC35C2 | solute carrier family 35 member C2 [Source:HGNC Symbol;Acc:HGNC:17117] | 2563 | 0.295 | 0.2060 | Yes |
| 74 | SLC15A3 | solute carrier family 15 member 3 [Source:HGNC Symbol;Acc:HGNC:18068] | 2612 | 0.290 | 0.2051 | Yes |
| 75 | NDUFB8 | NADH:ubiquinone oxidoreductase subunit B8 [Source:HGNC Symbol;Acc:HGNC:7703] | 2623 | 0.288 | 0.2072 | Yes |
| 76 | SLC35E3 | solute carrier family 35 member E3 [Source:HGNC Symbol;Acc:HGNC:20864] | 2624 | 0.287 | 0.2100 | Yes |
| 77 | SLC5A12 | solute carrier family 5 member 12 [Source:HGNC Symbol;Acc:HGNC:28750] | 2652 | 0.285 | 0.2107 | Yes |
| 78 | SLC17A5 | solute carrier family 17 member 5 [Source:HGNC Symbol;Acc:HGNC:10933] | 2671 | 0.283 | 0.2121 | Yes |
| 79 | ABCB6 | ATP binding cassette subfamily B member 6 (Langereis blood group) [Source:HGNC Symbol;Acc:HGNC:47] | 2717 | 0.278 | 0.2113 | Yes |
| 80 | SLC17A1 | solute carrier family 17 member 1 [Source:HGNC Symbol;Acc:HGNC:10929] | 2775 | 0.273 | 0.2095 | Yes |
| 81 | ATP2A1 | ATPase sarcoplasmic/endoplasmic reticulum Ca2+ transporting 1 [Source:HGNC Symbol;Acc:HGNC:811] | 2781 | 0.272 | 0.2118 | Yes |
| 82 | ATP6V0C | ATPase H+ transporting V0 subunit c [Source:HGNC Symbol;Acc:HGNC:855] | 2790 | 0.271 | 0.2139 | Yes |
| 83 | SLC9A7 | solute carrier family 9 member A7 [Source:HGNC Symbol;Acc:HGNC:17123] | 2817 | 0.268 | 0.2145 | Yes |
| 84 | ABCC8 | ATP binding cassette subfamily C member 8 [Source:HGNC Symbol;Acc:HGNC:59] | 2844 | 0.266 | 0.2151 | Yes |
| 85 | SLC16A2 | solute carrier family 16 member 2 [Source:HGNC Symbol;Acc:HGNC:10923] | 2852 | 0.265 | 0.2172 | Yes |
| 86 | SLC12A1 | solute carrier family 12 member 1 [Source:HGNC Symbol;Acc:HGNC:10910] | 2868 | 0.264 | 0.2186 | Yes |
| 87 | SLC16A4 | solute carrier family 16 member 4 [Source:HGNC Symbol;Acc:HGNC:10925] | 2876 | 0.263 | 0.2207 | Yes |
| 88 | COX6B1 | cytochrome c oxidase subunit 6B1 [Source:HGNC Symbol;Acc:HGNC:2280] | 2915 | 0.259 | 0.2202 | Yes |
| 89 | ABCG5 | ATP binding cassette subfamily G member 5 [Source:HGNC Symbol;Acc:HGNC:13886] | 2942 | 0.256 | 0.2207 | Yes |
| 90 | ATP2B2 | ATPase plasma membrane Ca2+ transporting 2 [Source:HGNC Symbol;Acc:HGNC:815] | 3023 | 0.247 | 0.2168 | Yes |
| 91 | ATP5F1B | ATP synthase F1 subunit beta [Source:HGNC Symbol;Acc:HGNC:830] | 3024 | 0.247 | 0.2193 | Yes |
| 92 | ABCD2 | ATP binding cassette subfamily D member 2 [Source:HGNC Symbol;Acc:HGNC:66] | 3031 | 0.246 | 0.2213 | Yes |
| 93 | SLC17A2 | solute carrier family 17 member 2 [Source:HGNC Symbol;Acc:HGNC:10930] | 3050 | 0.245 | 0.2223 | Yes |
| 94 | NDUFS2 | NADH:ubiquinone oxidoreductase core subunit S2 [Source:HGNC Symbol;Acc:HGNC:7708] | 3072 | 0.243 | 0.2230 | Yes |
| 95 | SLCO5A1 | solute carrier organic anion transporter family member 5A1 [Source:HGNC Symbol;Acc:HGNC:19046] | 3132 | 0.237 | 0.2207 | No |
| 96 | ATP4B | ATPase H+/K+ transporting subunit beta [Source:HGNC Symbol;Acc:HGNC:820] | 3160 | 0.234 | 0.2209 | No |
| 97 | SLC4A1 | solute carrier family 4 member 1 (Diego blood group) [Source:HGNC Symbol;Acc:HGNC:11027] | 3233 | 0.227 | 0.2175 | No |
| 98 | ATP2B3 | ATPase plasma membrane Ca2+ transporting 3 [Source:HGNC Symbol;Acc:HGNC:816] | 3263 | 0.225 | 0.2174 | No |
| 99 | SLC33A1 | solute carrier family 33 member 1 [Source:HGNC Symbol;Acc:HGNC:95] | 3320 | 0.219 | 0.2151 | No |
| 100 | SLC28A1 | solute carrier family 28 member 1 [Source:HGNC Symbol;Acc:HGNC:11001] | 3332 | 0.218 | 0.2164 | No |
| 101 | CTNS | "cystinosin, lysosomal cystine transporter [Source:HGNC Symbol;Acc:HGNC:2518]" | 3337 | 0.218 | 0.2183 | No |
| 102 | ABCC4 | ATP binding cassette subfamily C member 4 [Source:HGNC Symbol;Acc:HGNC:55] | 3344 | 0.217 | 0.2200 | No |
| 103 | COX7B | cytochrome c oxidase subunit 7B [Source:HGNC Symbol;Acc:HGNC:2291] | 3452 | 0.207 | 0.2135 | No |
| 104 | ABCA3 | ATP binding cassette subfamily A member 3 [Source:HGNC Symbol;Acc:HGNC:33] | 3549 | 0.198 | 0.2079 | No |
| 105 | SLCO1C1 | solute carrier organic anion transporter family member 1C1 [Source:HGNC Symbol;Acc:HGNC:13819] | 3566 | 0.197 | 0.2086 | No |
| 106 | ATP6V1G2 | ATPase H+ transporting V1 subunit G2 [Source:HGNC Symbol;Acc:HGNC:862] | 3709 | 0.184 | 0.1991 | No |
| 107 | COX15 | cytochrome c oxidase assembly homolog COX15 [Source:HGNC Symbol;Acc:HGNC:2263] | 3727 | 0.183 | 0.1996 | No |
| 108 | UQCRH | ubiquinol-cytochrome c reductase hinge protein [Source:HGNC Symbol;Acc:HGNC:12590] | 3827 | 0.174 | 0.1935 | No |
| 109 | ATP6V1F | ATPase H+ transporting V1 subunit F [Source:HGNC Symbol;Acc:HGNC:16832] | 3833 | 0.173 | 0.1948 | No |
| 110 | NDUFA6 | NADH:ubiquinone oxidoreductase subunit A6 [Source:HGNC Symbol;Acc:HGNC:7690] | 3834 | 0.173 | 0.1965 | No |
| 111 | ATP2B4 | ATPase plasma membrane Ca2+ transporting 4 [Source:HGNC Symbol;Acc:HGNC:817] | 3998 | 0.156 | 0.1852 | No |
| 112 | SLC38A4 | solute carrier family 38 member 4 [Source:HGNC Symbol;Acc:HGNC:14679] | 4000 | 0.156 | 0.1866 | No |
| 113 | SLC6A12 | solute carrier family 6 member 12 [Source:HGNC Symbol;Acc:HGNC:11045] | 4093 | 0.147 | 0.1808 | No |
| 114 | CLCN6 | chloride voltage-gated channel 6 [Source:HGNC Symbol;Acc:HGNC:2024] | 4138 | 0.143 | 0.1787 | No |
| 115 | SLC22A3 | solute carrier family 22 member 3 [Source:HGNC Symbol;Acc:HGNC:10967] | 4191 | 0.139 | 0.1760 | No |
| 116 | ABCC3 | ATP binding cassette subfamily C member 3 [Source:HGNC Symbol;Acc:HGNC:54] | 4200 | 0.138 | 0.1767 | No |
| 117 | SLC25A6 | solute carrier family 25 member 6 [Source:HGNC Symbol;Acc:HGNC:10992] | 4211 | 0.136 | 0.1773 | No |
| 118 | SLC9A1 | solute carrier family 9 member A1 [Source:HGNC Symbol;Acc:HGNC:11071] | 4221 | 0.135 | 0.1779 | No |
| 119 | SLC12A5 | solute carrier family 12 member 5 [Source:HGNC Symbol;Acc:HGNC:13818] | 4308 | 0.128 | 0.1724 | No |
| 120 | SLCO2B1 | solute carrier organic anion transporter family member 2B1 [Source:HGNC Symbol;Acc:HGNC:10962] | 4311 | 0.128 | 0.1735 | No |
| 121 | CLCN5 | chloride voltage-gated channel 5 [Source:HGNC Symbol;Acc:HGNC:2023] | 4372 | 0.123 | 0.1699 | No |
| 122 | SLC22A2 | solute carrier family 22 member 2 [Source:HGNC Symbol;Acc:HGNC:10966] | 4435 | 0.118 | 0.1662 | No |
| 123 | ABCA12 | ATP binding cassette subfamily A member 12 [Source:HGNC Symbol;Acc:HGNC:14637] | 4451 | 0.117 | 0.1662 | No |
| 124 | SLC5A4 | solute carrier family 5 member 4 [Source:HGNC Symbol;Acc:HGNC:11039] | 4472 | 0.115 | 0.1657 | No |
| 125 | ABCA8 | ATP binding cassette subfamily A member 8 [Source:HGNC Symbol;Acc:HGNC:38] | 4518 | 0.112 | 0.1633 | No |
| 126 | TAPBP | TAP binding protein [Source:HGNC Symbol;Acc:HGNC:11566] | 4527 | 0.111 | 0.1637 | No |
| 127 | SLC26A4 | solute carrier family 26 member 4 [Source:HGNC Symbol;Acc:HGNC:8818] | 4552 | 0.109 | 0.1629 | No |
| 128 | SLC6A14 | solute carrier family 6 member 14 [Source:HGNC Symbol;Acc:HGNC:11047] | 4634 | 0.101 | 0.1575 | No |
| 129 | ABCB7 | ATP binding cassette subfamily B member 7 [Source:HGNC Symbol;Acc:HGNC:48] | 4669 | 0.099 | 0.1558 | No |
| 130 | SLC5A7 | solute carrier family 5 member 7 [Source:HGNC Symbol;Acc:HGNC:14025] | 4701 | 0.096 | 0.1543 | No |
| 131 | SLCO4C1 | solute carrier organic anion transporter family member 4C1 [Source:HGNC Symbol;Acc:HGNC:23612] | 4714 | 0.095 | 0.1543 | No |
| 132 | ATP7A | ATPase copper transporting alpha [Source:HGNC Symbol;Acc:HGNC:869] | 4814 | 0.086 | 0.1473 | No |
| 133 | CYC1 | cytochrome c1 [Source:HGNC Symbol;Acc:HGNC:2579] | 4879 | 0.082 | 0.1430 | No |
| 134 | SLC4A4 | solute carrier family 4 member 4 [Source:HGNC Symbol;Acc:HGNC:11030] | 5027 | 0.071 | 0.1320 | No |
| 135 | SLC9A2 | solute carrier family 9 member A2 [Source:HGNC Symbol;Acc:HGNC:11072] | 5068 | 0.067 | 0.1295 | No |
| 136 | SLC25A4 | solute carrier family 25 member 4 [Source:HGNC Symbol;Acc:HGNC:10990] | 5073 | 0.067 | 0.1299 | No |
| 137 | SLC5A5 | solute carrier family 5 member 5 [Source:HGNC Symbol;Acc:HGNC:11040] | 5151 | 0.060 | 0.1244 | No |
| 138 | ABCC2 | ATP binding cassette subfamily C member 2 [Source:HGNC Symbol;Acc:HGNC:53] | 5206 | 0.056 | 0.1206 | No |
| 139 | COX6A2 | cytochrome c oxidase subunit 6A2 [Source:HGNC Symbol;Acc:HGNC:2279] | 5242 | 0.053 | 0.1184 | No |
| 140 | COX5A | cytochrome c oxidase subunit 5A [Source:HGNC Symbol;Acc:HGNC:2267] | 5329 | 0.045 | 0.1120 | No |
| 141 | SLC25A14 | solute carrier family 25 member 14 [Source:HGNC Symbol;Acc:HGNC:10984] | 5333 | 0.045 | 0.1122 | No |
| 142 | ABCA2 | ATP binding cassette subfamily A member 2 [Source:HGNC Symbol;Acc:HGNC:32] | 5349 | 0.044 | 0.1115 | No |
| 143 | ATP1A3 | ATPase Na+/K+ transporting subunit alpha 3 [Source:HGNC Symbol;Acc:HGNC:801] | 5475 | 0.035 | 0.1019 | No |
| 144 | UQCRFS1 | "ubiquinol-cytochrome c reductase, Rieske iron-sulfur polypeptide 1 [Source:HGNC Symbol;Acc:HGNC:12587]" | 5509 | 0.032 | 0.0996 | No |
| 145 | SLC8B1 | solute carrier family 8 member B1 [Source:HGNC Symbol;Acc:HGNC:26175] | 5524 | 0.031 | 0.0988 | No |
| 146 | SLC5A2 | solute carrier family 5 member 2 [Source:HGNC Symbol;Acc:HGNC:11037] | 5562 | 0.027 | 0.0961 | No |
| 147 | SLC6A2 | solute carrier family 6 member 2 [Source:HGNC Symbol;Acc:HGNC:11048] | 5750 | 0.012 | 0.0814 | No |
| 148 | ATP7B | ATPase copper transporting beta [Source:HGNC Symbol;Acc:HGNC:870] | 5751 | 0.012 | 0.0815 | No |
| 149 | CYB5A | cytochrome b5 type A [Source:HGNC Symbol;Acc:HGNC:2570] | 5850 | 0.005 | 0.0738 | No |
| 150 | SLC16A1 | solute carrier family 16 member 1 [Source:HGNC Symbol;Acc:HGNC:10922] | 6043 | -0.008 | 0.0586 | No |
| 151 | SLC16A8 | solute carrier family 16 member 8 [Source:HGNC Symbol;Acc:HGNC:16270] | 6248 | -0.024 | 0.0427 | No |
| 152 | ATP8A1 | ATPase phospholipid transporting 8A1 [Source:HGNC Symbol;Acc:HGNC:13531] | 6256 | -0.024 | 0.0423 | No |
| 153 | SLC12A8 | solute carrier family 12 member 8 [Source:HGNC Symbol;Acc:HGNC:15595] | 6318 | -0.029 | 0.0378 | No |
| 154 | ATP13A2 | ATPase cation transporting 13A2 [Source:HGNC Symbol;Acc:HGNC:30213] | 6336 | -0.031 | 0.0367 | No |
| 155 | TOMM20 | translocase of outer mitochondrial membrane 20 [Source:HGNC Symbol;Acc:HGNC:20947] | 6339 | -0.031 | 0.0369 | No |
| 156 | ABCB9 | ATP binding cassette subfamily B member 9 [Source:HGNC Symbol;Acc:HGNC:50] | 6442 | -0.038 | 0.0292 | No |
| 157 | SLC39A8 | solute carrier family 39 member 8 [Source:HGNC Symbol;Acc:HGNC:20862] | 6450 | -0.039 | 0.0290 | No |
| 158 | SLC4A8 | solute carrier family 4 member 8 [Source:HGNC Symbol;Acc:HGNC:11034] | 6455 | -0.039 | 0.0291 | No |
| 159 | SLC37A4 | solute carrier family 37 member 4 [Source:HGNC Symbol;Acc:HGNC:4061] | 6459 | -0.039 | 0.0292 | No |
| 160 | SLC7A11 | solute carrier family 7 member 11 [Source:HGNC Symbol;Acc:HGNC:11059] | 6470 | -0.040 | 0.0288 | No |
| 161 | SLC25A1 | solute carrier family 25 member 1 [Source:HGNC Symbol;Acc:HGNC:10979] | 6509 | -0.043 | 0.0263 | No |
| 162 | SLC15A1 | solute carrier family 15 member 1 [Source:HGNC Symbol;Acc:HGNC:10920] | 6515 | -0.043 | 0.0263 | No |
| 163 | ATP2A3 | ATPase sarcoplasmic/endoplasmic reticulum Ca2+ transporting 3 [Source:HGNC Symbol;Acc:HGNC:813] | 6559 | -0.046 | 0.0233 | No |
| 164 | SLC4A5 | solute carrier family 4 member 5 [Source:HGNC Symbol;Acc:HGNC:18168] | 6572 | -0.047 | 0.0229 | No |
| 165 | ABCG4 | ATP binding cassette subfamily G member 4 [Source:HGNC Symbol;Acc:HGNC:13884] | 6574 | -0.047 | 0.0232 | No |
| 166 | TAP1 | "transporter 1, ATP binding cassette subfamily B member [Source:HGNC Symbol;Acc:HGNC:43]" | 6633 | -0.052 | 0.0192 | No |
| 167 | SLC15A2 | solute carrier family 15 member 2 [Source:HGNC Symbol;Acc:HGNC:10921] | 6704 | -0.056 | 0.0142 | No |
| 168 | SLC17A7 | solute carrier family 17 member 7 [Source:HGNC Symbol;Acc:HGNC:16704] | 6773 | -0.061 | 0.0094 | No |
| 169 | NDUFA5 | NADH:ubiquinone oxidoreductase subunit A5 [Source:HGNC Symbol;Acc:HGNC:7688] | 6789 | -0.062 | 0.0088 | No |
| 170 | SLC22A4 | solute carrier family 22 member 4 [Source:HGNC Symbol;Acc:HGNC:10968] | 6912 | -0.073 | -0.0002 | No |
| 171 | SLC6A1 | solute carrier family 6 member 1 [Source:HGNC Symbol;Acc:HGNC:11042] | 6970 | -0.078 | -0.0039 | No |
| 172 | SLC1A2 | solute carrier family 1 member 2 [Source:HGNC Symbol;Acc:HGNC:10940] | 7026 | -0.082 | -0.0075 | No |
| 173 | SLC22A6 | solute carrier family 22 member 6 [Source:HGNC Symbol;Acc:HGNC:10970] | 7030 | -0.082 | -0.0069 | No |
| 174 | ATP6V1B2 | ATPase H+ transporting V1 subunit B2 [Source:HGNC Symbol;Acc:HGNC:854] | 7074 | -0.086 | -0.0094 | No |
| 175 | SLC45A2 | solute carrier family 45 member 2 [Source:HGNC Symbol;Acc:HGNC:16472] | 7144 | -0.092 | -0.0140 | No |
| 176 | SLC11A1 | solute carrier family 11 member 1 [Source:HGNC Symbol;Acc:HGNC:10907] | 7153 | -0.092 | -0.0137 | No |
| 177 | ABCB8 | ATP binding cassette subfamily B member 8 [Source:HGNC Symbol;Acc:HGNC:49] | 7303 | -0.102 | -0.0245 | No |
| 178 | SLC22A5 | solute carrier family 22 member 5 [Source:HGNC Symbol;Acc:HGNC:10969] | 7368 | -0.108 | -0.0285 | No |
| 179 | SLC6A5 | solute carrier family 6 member 5 [Source:HGNC Symbol;Acc:HGNC:11051] | 7439 | -0.114 | -0.0330 | No |
| 180 | SLC6A13 | solute carrier family 6 member 13 [Source:HGNC Symbol;Acc:HGNC:11046] | 7463 | -0.115 | -0.0336 | No |
| 181 | SLC16A10 | solute carrier family 16 member 10 [Source:HGNC Symbol;Acc:HGNC:17027] | 7469 | -0.116 | -0.0329 | No |
| 182 | SLC6A4 | solute carrier family 6 member 4 [Source:HGNC Symbol;Acc:HGNC:11050] | 7472 | -0.116 | -0.0319 | No |
| 183 | ABCG2 | ATP binding cassette subfamily G member 2 (Junior blood group) [Source:HGNC Symbol;Acc:HGNC:74] | 7473 | -0.116 | -0.0307 | No |
| 184 | SLC26A1 | solute carrier family 26 member 1 [Source:HGNC Symbol;Acc:HGNC:10993] | 7618 | -0.128 | -0.0409 | No |
| 185 | SLC17A3 | solute carrier family 17 member 3 [Source:HGNC Symbol;Acc:HGNC:10931] | 7650 | -0.131 | -0.0420 | No |
| 186 | ABCA6 | ATP binding cassette subfamily A member 6 [Source:HGNC Symbol;Acc:HGNC:36] | 7655 | -0.131 | -0.0410 | No |
| 187 | SLC36A1 | solute carrier family 36 member 1 [Source:HGNC Symbol;Acc:HGNC:18761] | 7670 | -0.132 | -0.0408 | No |
| 188 | SLC22A1 | solute carrier family 22 member 1 [Source:HGNC Symbol;Acc:HGNC:10963] | 7685 | -0.133 | -0.0406 | No |
| 189 | SLC17A4 | solute carrier family 17 member 4 [Source:HGNC Symbol;Acc:HGNC:10932] | 7743 | -0.137 | -0.0438 | No |
| 190 | ABCB11 | ATP binding cassette subfamily B member 11 [Source:HGNC Symbol;Acc:HGNC:42] | 7825 | -0.143 | -0.0488 | No |
| 191 | SLC25A5 | solute carrier family 25 member 5 [Source:HGNC Symbol;Acc:HGNC:10991] | 7877 | -0.146 | -0.0514 | No |
| 192 | SLC24A2 | solute carrier family 24 member 2 [Source:HGNC Symbol;Acc:HGNC:10976] | 7886 | -0.147 | -0.0505 | No |
| 193 | SLC16A5 | solute carrier family 16 member 5 [Source:HGNC Symbol;Acc:HGNC:10926] | 7947 | -0.151 | -0.0538 | No |
| 194 | ATP6V1A | ATPase H+ transporting V1 subunit A [Source:HGNC Symbol;Acc:HGNC:851] | 8013 | -0.156 | -0.0574 | No |
| 195 | SLC17A6 | solute carrier family 17 member 6 [Source:HGNC Symbol;Acc:HGNC:16703] | 8119 | -0.164 | -0.0641 | No |
| 196 | SLC1A1 | solute carrier family 1 member 1 [Source:HGNC Symbol;Acc:HGNC:10939] | 8157 | -0.167 | -0.0654 | No |
| 197 | ATP1A2 | ATPase Na+/K+ transporting subunit alpha 2 [Source:HGNC Symbol;Acc:HGNC:800] | 8218 | -0.173 | -0.0684 | No |
| 198 | ABCB1 | ATP binding cassette subfamily B member 1 [Source:HGNC Symbol;Acc:HGNC:40] | 8228 | -0.173 | -0.0674 | No |
| 199 | SLC13A2 | solute carrier family 13 member 2 [Source:HGNC Symbol;Acc:HGNC:10917] | 8259 | -0.176 | -0.0680 | No |
| 200 | ATP1A1 | ATPase Na+/K+ transporting subunit alpha 1 [Source:HGNC Symbol;Acc:HGNC:799] | 8282 | -0.177 | -0.0680 | No |
| 201 | SLC35D1 | solute carrier family 35 member D1 [Source:HGNC Symbol;Acc:HGNC:20800] | 8408 | -0.186 | -0.0761 | No |
| 202 | SLC10A1 | solute carrier family 10 member 1 [Source:HGNC Symbol;Acc:HGNC:10905] | 8432 | -0.188 | -0.0760 | No |
| 203 | SLC20A2 | solute carrier family 20 member 2 [Source:HGNC Symbol;Acc:HGNC:10947] | 8459 | -0.190 | -0.0762 | No |
| 204 | SLC1A7 | solute carrier family 1 member 7 [Source:HGNC Symbol;Acc:HGNC:10945] | 8504 | -0.194 | -0.0778 | No |
| 205 | SLC24A1 | solute carrier family 24 member 1 [Source:HGNC Symbol;Acc:HGNC:10975] | 8551 | -0.198 | -0.0794 | No |
| 206 | SLC25A10 | solute carrier family 25 member 10 [Source:HGNC Symbol;Acc:HGNC:10980] | 8552 | -0.198 | -0.0775 | No |
| 207 | ABCD4 | ATP binding cassette subfamily D member 4 [Source:HGNC Symbol;Acc:HGNC:68] | 8652 | -0.205 | -0.0833 | No |
| 208 | SLC25A3 | solute carrier family 25 member 3 [Source:HGNC Symbol;Acc:HGNC:10989] | 8681 | -0.208 | -0.0834 | No |
| 209 | SLC25A22 | solute carrier family 25 member 22 [Source:HGNC Symbol;Acc:HGNC:19954] | 8682 | -0.208 | -0.0814 | No |
| 210 | SLC6A9 | solute carrier family 6 member 9 [Source:HGNC Symbol;Acc:HGNC:11056] | 8704 | -0.209 | -0.0809 | No |
| 211 | SLC35D2 | solute carrier family 35 member D2 [Source:HGNC Symbol;Acc:HGNC:20799] | 8714 | -0.210 | -0.0796 | No |
| 212 | SLC23A2 | solute carrier family 23 member 2 [Source:HGNC Symbol;Acc:HGNC:10973] | 8732 | -0.211 | -0.0788 | No |
| 213 | SLC20A1 | solute carrier family 20 member 1 [Source:HGNC Symbol;Acc:HGNC:10946] | 8787 | -0.215 | -0.0810 | No |
| 214 | CLCN7 | chloride voltage-gated channel 7 [Source:HGNC Symbol;Acc:HGNC:2025] | 8795 | -0.216 | -0.0794 | No |
| 215 | SLC4A2 | solute carrier family 4 member 2 [Source:HGNC Symbol;Acc:HGNC:11028] | 8828 | -0.220 | -0.0797 | No |
| 216 | ABCA7 | ATP binding cassette subfamily A member 7 [Source:HGNC Symbol;Acc:HGNC:37] | 8856 | -0.222 | -0.0796 | No |
| 217 | ABCD3 | ATP binding cassette subfamily D member 3 [Source:HGNC Symbol;Acc:HGNC:67] | 8877 | -0.223 | -0.0790 | No |
| 218 | SLC12A4 | solute carrier family 12 member 4 [Source:HGNC Symbol;Acc:HGNC:10913] | 8893 | -0.224 | -0.0780 | No |
| 219 | SLC12A7 | solute carrier family 12 member 7 [Source:HGNC Symbol;Acc:HGNC:10915] | 8904 | -0.225 | -0.0765 | No |
| 220 | SLC34A1 | solute carrier family 34 member 1 [Source:HGNC Symbol;Acc:HGNC:11019] | 8919 | -0.226 | -0.0754 | No |
| 221 | ABCD1 | ATP binding cassette subfamily D member 1 [Source:HGNC Symbol;Acc:HGNC:61] | 8930 | -0.226 | -0.0739 | No |
| 222 | SLC13A1 | solute carrier family 13 member 1 [Source:HGNC Symbol;Acc:HGNC:10916] | 8963 | -0.229 | -0.0742 | No |
| 223 | SLC13A4 | solute carrier family 13 member 4 [Source:HGNC Symbol;Acc:HGNC:15827] | 9004 | -0.232 | -0.0750 | No |
| 224 | ATP2A2 | ATPase sarcoplasmic/endoplasmic reticulum Ca2+ transporting 2 [Source:HGNC Symbol;Acc:HGNC:812] | 9015 | -0.233 | -0.0735 | No |
| 225 | ABCC5 | ATP binding cassette subfamily C member 5 [Source:HGNC Symbol;Acc:HGNC:56] | 9066 | -0.237 | -0.0751 | No |
| 226 | SLC11A2 | solute carrier family 11 member 2 [Source:HGNC Symbol;Acc:HGNC:10908] | 9152 | -0.244 | -0.0794 | No |
| 227 | ABCC10 | ATP binding cassette subfamily C member 10 [Source:HGNC Symbol;Acc:HGNC:52] | 9218 | -0.250 | -0.0821 | No |
| 228 | SLC18A1 | solute carrier family 18 member A1 [Source:HGNC Symbol;Acc:HGNC:10934] | 9252 | -0.252 | -0.0822 | No |
| 229 | SLC26A3 | solute carrier family 26 member 3 [Source:HGNC Symbol;Acc:HGNC:3018] | 9261 | -0.253 | -0.0803 | No |
| 230 | ABCC9 | ATP binding cassette subfamily C member 9 [Source:HGNC Symbol;Acc:HGNC:60] | 9346 | -0.259 | -0.0844 | No |
| 231 | KCNJ8 | potassium inwardly rectifying channel subfamily J member 8 [Source:HGNC Symbol;Acc:HGNC:6269] | 9421 | -0.266 | -0.0877 | No |
| 232 | SLC26A2 | solute carrier family 26 member 2 [Source:HGNC Symbol;Acc:HGNC:10994] | 9523 | -0.276 | -0.0929 | No |
| 233 | CYBRD1 | cytochrome b reductase 1 [Source:HGNC Symbol;Acc:HGNC:20797] | 9585 | -0.282 | -0.0950 | No |
| 234 | SLC22A8 | solute carrier family 22 member 8 [Source:HGNC Symbol;Acc:HGNC:10972] | 9645 | -0.287 | -0.0968 | No |
| 235 | SLC1A3 | solute carrier family 1 member 3 [Source:HGNC Symbol;Acc:HGNC:10941] | 9656 | -0.288 | -0.0947 | No |
| 236 | ABCB4 | ATP binding cassette subfamily B member 4 [Source:HGNC Symbol;Acc:HGNC:45] | 9807 | -0.302 | -0.1036 | No |
| 237 | ABCA1 | ATP binding cassette subfamily A member 1 [Source:HGNC Symbol;Acc:HGNC:29] | 9874 | -0.309 | -0.1058 | No |
| 238 | SLC8A2 | solute carrier family 8 member A2 [Source:HGNC Symbol;Acc:HGNC:11069] | 9887 | -0.310 | -0.1036 | No |
| 239 | SLC26A6 | solute carrier family 26 member 6 [Source:HGNC Symbol;Acc:HGNC:14472] | 9898 | -0.311 | -0.1013 | No |
| 240 | SLC19A1 | solute carrier family 19 member 1 [Source:HGNC Symbol;Acc:HGNC:10937] | 9931 | -0.315 | -0.1007 | No |
| 241 | ATP13A1 | ATPase 13A1 [Source:HGNC Symbol;Acc:HGNC:24215] | 9939 | -0.315 | -0.0981 | No |
| 242 | SLC6A16 | solute carrier family 6 member 16 [Source:HGNC Symbol;Acc:HGNC:13622] | 9998 | -0.321 | -0.0995 | No |
| 243 | SLCO1B3 | solute carrier organic anion transporter family member 1B3 [Source:HGNC Symbol;Acc:HGNC:10961] | 10011 | -0.323 | -0.0973 | No |
| 244 | SLC13A3 | solute carrier family 13 member 3 [Source:HGNC Symbol;Acc:HGNC:14430] | 10096 | -0.330 | -0.1007 | No |
| 245 | SLC16A6 | solute carrier family 16 member 6 [Source:HGNC Symbol;Acc:HGNC:10927] | 10128 | -0.334 | -0.0998 | No |
| 246 | SLC9A8 | solute carrier family 9 member A8 [Source:HGNC Symbol;Acc:HGNC:20728] | 10170 | -0.338 | -0.0997 | No |
| 247 | SLC6A3 | solute carrier family 6 member 3 [Source:HGNC Symbol;Acc:HGNC:11049] | 10177 | -0.338 | -0.0968 | No |
| 248 | SLC6A7 | solute carrier family 6 member 7 [Source:HGNC Symbol;Acc:HGNC:11054] | 10185 | -0.339 | -0.0940 | No |
| 249 | SLC10A2 | solute carrier family 10 member 2 [Source:HGNC Symbol;Acc:HGNC:10906] | 10202 | -0.341 | -0.0918 | No |
| 250 | SLC7A8 | solute carrier family 7 member 8 [Source:HGNC Symbol;Acc:HGNC:11066] | 10228 | -0.344 | -0.0904 | No |
| 251 | SLC39A14 | solute carrier family 39 member 14 [Source:HGNC Symbol;Acc:HGNC:20858] | 10367 | -0.358 | -0.0978 | No |
| 252 | ATP4A | ATPase H+/K+ transporting subunit alpha [Source:HGNC Symbol;Acc:HGNC:819] | 10417 | -0.363 | -0.0981 | No |
| 253 | SLC47A1 | solute carrier family 47 member 1 [Source:HGNC Symbol;Acc:HGNC:25588] | 10423 | -0.363 | -0.0948 | No |
| 254 | ATP2C1 | ATPase secretory pathway Ca2+ transporting 1 [Source:HGNC Symbol;Acc:HGNC:13211] | 10432 | -0.364 | -0.0918 | No |
| 255 | SLC38A3 | solute carrier family 38 member 3 [Source:HGNC Symbol;Acc:HGNC:18044] | 10443 | -0.366 | -0.0890 | No |
| 256 | SLCO2A1 | solute carrier organic anion transporter family member 2A1 [Source:HGNC Symbol;Acc:HGNC:10955] | 10546 | -0.378 | -0.0933 | No |
| 257 | ABCA4 | ATP binding cassette subfamily A member 4 [Source:HGNC Symbol;Acc:HGNC:34] | 10554 | -0.379 | -0.0901 | No |
| 258 | ATP2C2 | ATPase secretory pathway Ca2+ transporting 2 [Source:HGNC Symbol;Acc:HGNC:29103] | 10580 | -0.382 | -0.0883 | No |
| 259 | SLC38A1 | solute carrier family 38 member 1 [Source:HGNC Symbol;Acc:HGNC:13447] | 10617 | -0.385 | -0.0873 | No |
| 260 | SLC1A6 | solute carrier family 1 member 6 [Source:HGNC Symbol;Acc:HGNC:10944] | 10626 | -0.386 | -0.0841 | No |
| 261 | CDH17 | cadherin 17 [Source:HGNC Symbol;Acc:HGNC:1756] | 10667 | -0.390 | -0.0834 | No |
| 262 | SLC12A6 | solute carrier family 12 member 6 [Source:HGNC Symbol;Acc:HGNC:10914] | 10876 | -0.413 | -0.0958 | No |
| 263 | SLC28A3 | solute carrier family 28 member 3 [Source:HGNC Symbol;Acc:HGNC:16484] | 10910 | -0.418 | -0.0942 | No |
| 264 | CFTR | CF transmembrane conductance regulator [Source:HGNC Symbol;Acc:HGNC:1884] | 10920 | -0.420 | -0.0908 | No |
| 265 | SLC16A3 | solute carrier family 16 member 3 [Source:HGNC Symbol;Acc:HGNC:10924] | 10962 | -0.425 | -0.0898 | No |
| 266 | MFSD12 | major facilitator superfamily domain containing 12 [Source:HGNC Symbol;Acc:HGNC:28299] | 10965 | -0.426 | -0.0857 | No |
| 267 | SLC6A15 | solute carrier family 6 member 15 [Source:HGNC Symbol;Acc:HGNC:13621] | 10966 | -0.426 | -0.0814 | No |
| 268 | SLC6A20 | solute carrier family 6 member 20 [Source:HGNC Symbol;Acc:HGNC:30927] | 10982 | -0.428 | -0.0784 | No |
| 269 | ATP13A3 | ATPase 13A3 [Source:HGNC Symbol;Acc:HGNC:24113] | 11136 | -0.448 | -0.0861 | No |
| 270 | SLC35C1 | solute carrier family 35 member C1 [Source:HGNC Symbol;Acc:HGNC:20197] | 11286 | -0.470 | -0.0932 | No |
| 271 | SLC4A3 | solute carrier family 4 member 3 [Source:HGNC Symbol;Acc:HGNC:11029] | 11318 | -0.474 | -0.0909 | No |
| 272 | SLC25A16 | solute carrier family 25 member 16 [Source:HGNC Symbol;Acc:HGNC:10986] | 11411 | -0.488 | -0.0934 | No |
| 273 | SLC6A8 | solute carrier family 6 member 8 [Source:HGNC Symbol;Acc:HGNC:11055] | 11453 | -0.496 | -0.0917 | No |
| 274 | ABCC1 | ATP binding cassette subfamily C member 1 [Source:HGNC Symbol;Acc:HGNC:51] | 11462 | -0.497 | -0.0874 | No |
| 275 | SLC12A3 | solute carrier family 12 member 3 [Source:HGNC Symbol;Acc:HGNC:10912] | 11542 | -0.506 | -0.0886 | No |
| 276 | SLCO3A1 | solute carrier organic anion transporter family member 3A1 [Source:HGNC Symbol;Acc:HGNC:10952] | 11564 | -0.510 | -0.0852 | No |
| 277 | SLC5A1 | solute carrier family 5 member 1 [Source:HGNC Symbol;Acc:HGNC:11036] | 11582 | -0.512 | -0.0814 | No |
| 278 | SLC4A7 | solute carrier family 4 member 7 [Source:HGNC Symbol;Acc:HGNC:11033] | 11587 | -0.513 | -0.0766 | No |
| 279 | TAP2 | "transporter 2, ATP binding cassette subfamily B member [Source:HGNC Symbol;Acc:HGNC:44]" | 11616 | -0.519 | -0.0737 | No |
| 280 | ATP2B1 | ATPase plasma membrane Ca2+ transporting 1 [Source:HGNC Symbol;Acc:HGNC:814] | 11648 | -0.524 | -0.0709 | No |
| 281 | SLC22A7 | solute carrier family 22 member 7 [Source:HGNC Symbol;Acc:HGNC:10971] | 11678 | -0.528 | -0.0680 | No |
| 282 | SLC38A2 | solute carrier family 38 member 2 [Source:HGNC Symbol;Acc:HGNC:13448] | 11722 | -0.536 | -0.0660 | No |
| 283 | SLC12A2 | solute carrier family 12 member 2 [Source:HGNC Symbol;Acc:HGNC:10911] | 11744 | -0.540 | -0.0623 | No |
| 284 | SLC5A6 | solute carrier family 5 member 6 [Source:HGNC Symbol;Acc:HGNC:11041] | 11809 | -0.551 | -0.0619 | No |
| 285 | SLC22A11 | solute carrier family 22 member 11 [Source:HGNC Symbol;Acc:HGNC:18120] | 11826 | -0.554 | -0.0577 | No |
| 286 | ABCA5 | ATP binding cassette subfamily A member 5 [Source:HGNC Symbol;Acc:HGNC:35] | 11884 | -0.568 | -0.0565 | No |
| 287 | SLC6A11 | solute carrier family 6 member 11 [Source:HGNC Symbol;Acc:HGNC:11044] | 11916 | -0.575 | -0.0533 | No |
| 288 | SLC6A6 | solute carrier family 6 member 6 [Source:HGNC Symbol;Acc:HGNC:11052] | 12128 | -0.626 | -0.0638 | No |
| 289 | SLC8A1 | solute carrier family 8 member A1 [Source:HGNC Symbol;Acc:HGNC:11068] | 12192 | -0.642 | -0.0624 | No |
| 290 | SLC7A5 | solute carrier family 7 member 5 [Source:HGNC Symbol;Acc:HGNC:11063] | 12203 | -0.646 | -0.0567 | No |
| 291 | LETM1 | leucine zipper and EF-hand containing transmembrane protein 1 [Source:HGNC Symbol;Acc:HGNC:6556] | 12272 | -0.669 | -0.0555 | No |
| 292 | SLC35E2A | solute carrier family 35 member E2A [Source:HGNC Symbol;Acc:HGNC:20863] | 12330 | -0.686 | -0.0532 | No |
| 293 | SLC1A5 | solute carrier family 1 member 5 [Source:HGNC Symbol;Acc:HGNC:10943] | 12501 | -0.748 | -0.0592 | No |
| 294 | SLC16A7 | solute carrier family 16 member 7 [Source:HGNC Symbol;Acc:HGNC:10928] | 12545 | -0.771 | -0.0549 | No |
| 295 | SLC7A6 | solute carrier family 7 member 6 [Source:HGNC Symbol;Acc:HGNC:11064] | 12551 | -0.774 | -0.0476 | No |
| 296 | SLC3A2 | solute carrier family 3 member 2 [Source:HGNC Symbol;Acc:HGNC:11026] | 12587 | -0.789 | -0.0425 | No |
| 297 | SLCO4A1 | solute carrier organic anion transporter family member 4A1 [Source:HGNC Symbol;Acc:HGNC:10953] | 12593 | -0.792 | -0.0350 | No |
| 298 | SLC9A5 | solute carrier family 9 member A5 [Source:HGNC Symbol;Acc:HGNC:11078] | 12642 | -0.820 | -0.0307 | No |
| 299 | SLC28A2 | solute carrier family 28 member 2 [Source:HGNC Symbol;Acc:HGNC:11002] | 12660 | -0.833 | -0.0237 | No |
| 300 | SLC37A1 | solute carrier family 37 member 1 [Source:HGNC Symbol;Acc:HGNC:11024] | 12736 | -0.899 | -0.0207 | No |
| 301 | MT-ND5 | mitochondrially encoded NADH:ubiquinone oxidoreductase core subunit 5 [Source:HGNC Symbol;Acc:HGNC:7461] | 12743 | -0.906 | -0.0122 | No |
| 302 | SLC35E1 | solute carrier family 35 member E1 [Source:HGNC Symbol;Acc:HGNC:20803] | 12806 | -0.981 | -0.0073 | No |
| 303 | SLC26A10 | solute carrier family 26 member 10 [Source:HGNC Symbol;Acc:HGNC:14470] | 12899 | -1.475 | 0.0001 | No |
Table: GSEA details [plain text format]

  

Fig 2: GOMF\_ACTIVE\_TRANSMEMBRANE\_TRANSPORTER\_ACTIVITY      
 Blue-Pink O' Gram in the Space of the Analyzed GeneSet

  

Fig 3: GOMF\_ACTIVE\_TRANSMEMBRANE\_TRANSPORTER\_ACTIVITY: Random ES distribution      
 Gene set null distribution of ES for **GOMF\_ACTIVE\_TRANSMEMBRANE\_TRANSPORTER\_ACTIVITY**
